# The homeland of Proto-Tungusic inferred from contemporary words and ancient genomes

**DOI:** 10.1017/ehs.2020.8

**Published:** 2020-04-22

**Authors:** Chuan-Chao Wang, Martine Robbeets

**Affiliations:** 1 Department of Anthropology and Ethnology, Institute of Anthropology, National Institute for Data Science in Health and Medicine, and School of Life Sciences, Xiamen University, Xiamen 361005, China; 2 Eurasia3angle Research Group, Max Planck Institute for the Science of Human History, Jena, Germany

**Keywords:** archaeolinguistics, ancient DNA, Tungusic language family, homeland, Amur genome

## Abstract

The Tungusic languages form a language family spoken in Xinjiang, Siberia, Manchuria and the Russian Far East. There is a general consensus that these languages are genealogically related and descend from a common ancestral language, conventionally called ‘Proto-Tungusic’. However, the exact geographical location where the ancestral speakers of Proto-Tungusic originated from is subject to debate. Here we take an unprecedented approach to this problem, by integrating linguistic, archaeological and genetic evidence in a single study. Our analysis of ancient DNA suggests genetic continuity between an ancient Amur genetic lineage and the contemporary speakers of the Tungusic languages. Adding an archaeolinguistic perspective, we infer that the most plausible homeland for the speakers of Proto-Tungusic is the region around Lake Khanka in the Russian Far East. Our study pushes the field forward in answering the tantalizing question about the location of the Tungusic homeland and in illustrating how these three disciplines can converge into a holistic approach to the human past.

## Graphical abstract

**Figure:**
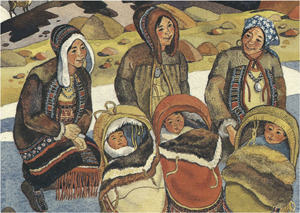

**Media summary:** The Tungusic languages form a language family spoken in Xinjiang, Siberia, Manchuria and the Russian Far East. There is a general consensus that these languages descend from a common ancestral language, conventionally called ‘Proto-Tungusic’. However, the exact geographical location where the ancestral speakers of Proto-Tungusic originated from is subject to debate. Integrating linguistic, archaeological and genetic evidence in a single study, we here show that the most plausible homeland for the speakers of Proto-Tungusic is the region around Lake Khanka in the Russian Far East. Our study pushes the field forward in answering the tantalizing question about the location of the Tungusic homeland and in illustrating how these three disciplines can converge into a holistic approach on the human past.

## Introduction

Even if they are rapidly losing ground owing to Chinese and Russian influence, the speakers of Tungusic languages are widely distributed all over Northeast Asia, including Xinjiang, Siberia, Manchuria and the Russian Far East. Their wide distribution and the considerable internal variation of populations have made the exact geographical location where the ancestral speakers of Proto-Tungusic originated from subject to debate. Traditionally, this issue was approached through the combined investigation of languages and material remains, but our recently acquired ability to sequence DNA directly from ancient human remains has considerably expanded the available scientific tool kit. Therefore, here we intend to complement linguistic and archaeological observations with genetic evidence to infer the most plausible homeland for the speakers of Proto-Tungusic.

**Figure 1.**
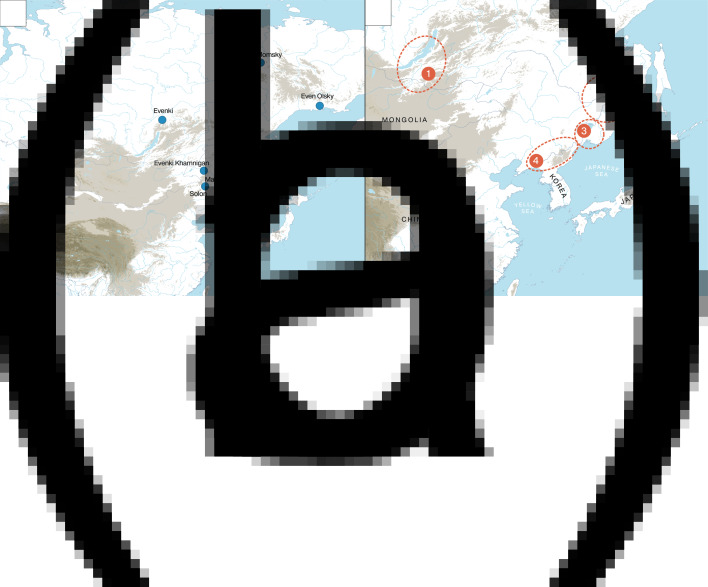
(a) The distribution of the Tungusic languages; (b) proposed locations for the homeland of the ancestral speakers of Proto-Tungusic.

The contemporary and historically attested Tungusic languages are spread from the Okhotsk Sea in the east to the Yenisei Basin in the west, and from the Bohai Sea in the south to the Arctic Ocean in the north. Figure 1 shows the distribution of 12 Tungusic languages, Oroch, Udihe, Hezhe, Nanai, Orok, Olcha, Xibo, Even, Solon, Evenki, Negidal and Oroqen, along with dialectal varieties such as Kamnigan Evenki, Momsky Even, Olsky Even, Najkhin Nanai, Kur-Urmi Nanai and Bikin Nanai as well as two historical varieties, Jurchen and Manchu. Since written materials in Jurchen, the now-extinct language of the Jin dynasty (AD 1115–1234) are only partially deciphered, the earliest well-documented stage is Manchu, the official language of the Qing dynasty (AD 1644–1911).

Although there is a general consensus that these languages are genealogically related and descend from a common ancestral language, conventionally called ‘Proto-Tungusic’, there are various competing hypotheses concerning the possible homeland of this language. Among the proposals, we find (a) the Baikal region (Vasilevich 1960; Menges 1968; Derevyanko 1976; Helimski 1985), (b) the Mid Amur and the lower part of the Upper Amur region (Janhunen 1996: 169; Korovina 2011; Pevnov 2012; Wichmann, personal communication, 3 October 2019), (c) the Ussuri region around Lake Khanka, a tributary of the Amur (Robbeets *et al*. 2020) and (d) the Yalu River region on the border between present-day Liaoning and Northern Korea (Janhunen 2012). These locations are situated on the map in Figure 1.

After assessing these four different hypotheses from an archaeolinguistic perspective, we will open a genetic window into this question. By supplementing our archaeolinguistic argumentation with genetic evidence for continuity between an ancient Amur genetic profile and contemporary speakers of Tungusic, we will conclude that the region around Lake Khanka constitutes the most plausible homeland for the speakers of Proto-Tungusic. Through this holistic approach, we hope to answer the tantalizing question about the Tungusic homeland, posed by numerous specialists in the past.

## Methods

### Archaeolinguistic methods

Traditionally, there are two principal techniques for attempting to determine the location of the original homeland of the speakers of a certain proto-language: the diversity hotspot principle and cultural reconstruction. The ‘diversity hotspot principle’ is a loose principle that was originally introduced by Edward Sapir (1916: 87) as the ‘center of gravity principle’, but it is also known as the ‘focus of diversity’ principle (Heggarty 2015: 612–613). Assuming that the deepest splits within a family reflect the greatest age, the location of these splits on the map is thought to point to the area where the proto-language began to diversify. The principle is thus based on the assumption that the homeland is closest to where one finds the greatest diversity with regard to the deepest subgroups of the language family. Attempts have been made to formalize this principle in a computer-automated approach, computing a diversity measure for each language and identifying the homeland with the location of the language with the highest diversity measure (Wichmann *et al*. 2010). A schoolbook example is the Austronesian family, that extends across a huge geographical range, all the way from Madagascar to Easter Island, but the deepest subgroups are found on just one Island, Taiwan, which is therefore identified as the homeland.

Although the diversity hotspot principle can provide some clues about the homeland of a language family, it must also contend with certain limitations. First, the identification of the homeland depends on the location of the deepest subgroups and therefore on how robustly the internal structure of a given family has been established. A second limitation of this principle is that the contemporary hotspot of linguistic diversity may diverge from the earlier one. Looking at the present map of Indo-European with the Balkan Peninsula hosting the highest diversity of deep subgroups, we might conclude that the homeland is there, instead of the Pontic Steppe or Anatolia. This example makes it clear that the application of the diversity hotspot principle at more profound time-depths may be speculative because earlier diversity may have been erased through time. Third, the diversity hotspot principle might also be upset when a migration was suddenly directed over a long distance rather than representing slow, gradual and random movement into adjacent areas. Finally, linguistic diversity is a function not only of time but also of other factors such as environmental change and disease. These may have made the original homeland unsuitable for human habitation at a certain point in time. In this way, original linguistic diversity may have been erased and it may no longer be possible to pinpoint the homeland using the diversity hotspot principle. However, even if the principle is not foolproof, it offers valuable clues for the location of a homeland at less remote time-depths. In the case of Tungusic, there is only mild disagreement on the primary branching structure, while the time depth is agreed to be relatively shallow, i.e. between 600 BCE and AD 600. Except for the historical transfer of the Xibo tribe speaking Manchu in 1763 from Manchuria to Xinjiang, there are no known cases of sudden long-distance movement. Therefore, we can gain more from applying the diversity hotspot principle to Tungusic than we can lose from ignoring it.

The second technique, cultural reconstruction, looks at the cultural vocabulary revealed in the reconstructed vocabulary of a proto-language. The method was first introduced under the label ‘linguistic paleontology’ by Adolphe Pictet (1859), but it became widespread under the banner of the ‘Wörter und Sachen’ movement, which used reconstructed proto-words as a way of determining proto-culture (Shuchardt 1912). Cultural reconstruction is a vocabulary-based approach that enables us to study human prehistory by reconstructing lexical items that are ecologically or culturally diagnostic for a particular region at a particular time. Clues for fauna and flora can help to delimit a homeland if several reconstructed plant and animal names converge in a particular region to the exclusion of other regions. Reconstruction of cultural items such as cultivars and feedstock can help to identify an archaeological culture yielding the relevant material remains. The method can be combined with diachronic contact linguistics, the study of the ways in which ancestral languages influenced each other when their speakers interacted. As shown by the assumed borrowing of the term ‘barley’ below, this approach can be useful to shed light on prehistoric ethnic interaction and help us determine the chronology of our data.

Among the limitations of cultural reconstruction are the potential lack of accuracy in semantic reconstruction, the occurrence of lexical recycling, the deception of a single item not backed up by a semantic domain and the shakiness of inferences made on the basis of absence. Since semantic reconstruction is less precise than phonological reconstruction, we should first be cautious that the meaning assigned to a reconstructed form can be no more specific than the meaning shared by all the cognate forms.

Second, we should be aware of the possibility of ‘lexical recycling’, a process whereby words with a general, non-cultural meaning become repurposed as words with a specific, cultural meaning after the importation or invention of the corresponding innovation. The reconstruction for ‘foxtail millet’ in Supplementary Table S1 (3), for instance, could also indicate the wild variety because the crop is native to the Russian Far East.

Third, we should avoid reconstructing a single cultural item that is not backed up by other members of the semantic domain. Paying attention to the clustering of different cultural reconstructions in a specific semantic domain, such as agriculture, can contribute to a fuller picture of prehistory than the study of individual cultural reconstructions.

And fourth, ‘absence of evidence is not evidence of absence’. The observation that an agricultural lexicon cannot be reconstructed for a certain proto-language may be explained by the fact that the proto-speakers simply were not familiar with farming, but it could also be due to the lack of exhaustive research or to the attrition of agricultural cognates over time. Therefore, inferences made on the basis of absence are not necessarily wrong, but they should not be taken as absolute proof for an argument.

Nevertheless, when combined with the necessary checks and balances, cultural reconstruction is reasonably robust as a way of determining proto-ecology and proto-culture through proto-language. Therefore, we will apply this method in order to make predictions about the cultural and natural environment available to the speakers of Proto-Tungusic.

### Genetic methods

We reanalyzed the genome-wide data of ancient and present-day East Eurasian populations related to Tungusic-speaking populations from the following literature: Siska *et al*. (2017), de Barros Damgaard *et al*. (2018), Kanzawa-Kiriyama *et al*. (2016), Patterson *et al*. (2012), Jeong *et al*. (2019) and Lazaridis *et al*. (2014).

We first carried out principal components analysis in the smartpca program of EIGENSOFT (Patterson *et al*. 2006), using default parameters and the lsqproject: YES and numoutlieriter: 0 options. We projected ancient Devil's Gate Ustlda and Jomon samples onto the variation of present-day East Eurasians from the published Human Origin Dataset over 591,642 SNPs. We carried out ADMIXTURE analysis after pruning for linkage disequilibrium in PLINK with parameters --indep-pairwise 200 25 0.4 Chang *et al.* (2015) which retained 332,959 SNPs. We ran ADMIXTURE (Alexander *et al*. 2009) with default 5-fold cross-validation (--cv = 5), varying the number of ancestral populations between *K* = 2 and *K* = 12 in 100 bootstraps with different random seeds. We observed the lowest CV error at *K* = 8. The samples used in this analysis are present-day East Eurasians, and individuals from worldwide representative populations. We used outgroup *f*_3_-statistics of the form *f*_3_ (Mbuti; X, Y) to test the genetic drift sharing between ancient Devil's Gate and present-day East Eurasians. Analysis of *f*_3_-statistics was carried out using ADMIXTOOLS with standard errors computed with a block jackknife (Patterson *et al*. 2012). We used *qpAdm* as implemented in ADMIXTOOLS to estimate West Eurasian mixture proportions in Even and Evenk with a set of outgroup populations. Weighted linkage disequilibrium (LD) decay was calculated using ALDER to infer admixture parameters including dates and mixture proportions (Loh *et al*. 2013). We added Table S2 representing West Eurasian-related admixture proportions estimated using *qpAdm* and Table S3 listing the admixture time with West Eurasian-related groups estimated by ALDER in the Supplementary Information.

## Results

### Archaeolinguistic results

**Figure 2.**
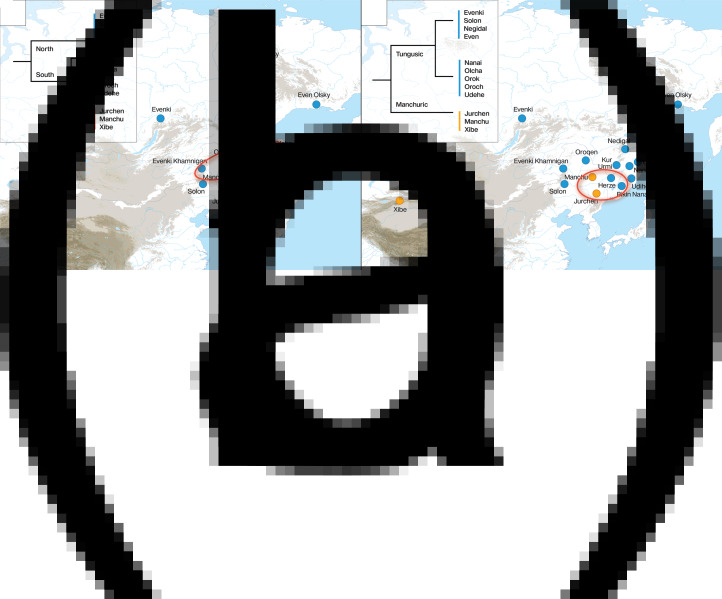
Diversity hotspot of the Tungusic languages under the north–south classification in (a) as compared with the hotspot under the Manchu–Tungusic classification in (b).

The principle that the geographical area of maximal primary diversity within a language family corresponds to the original homeland implies that the primary splits in the family are determinant for the location of the homeland. There is some disagreement on the internal structure of the Tungusic language family, mainly with respect to the separation of the Manchuric branch. As shown in Figure 2, some authors proposed a north–south classification, in which the separation of Manchuric from the other Tungusic languages does not constitute the earliest split in the family (Cincius 1949; Benzing 1955; Kormušin 1998; Georg 2004; Janhunen 2012), while others proposed an early breakup between Manchuric and the rest of Tungusic (Sunik 1959; Vasilevich 1960; Doerfer 1978; Vovin 1993; Robbeets 2015; Whaley and Oskolskaya 2020). Depending on which of both classifications of Tungusic we favor, the center of primary diversity – and thus also the inferred location of the homeland – will move on the map, as illustrated in Figure 2. Whereas the north–south classification pushes the homeland to the north into the Mid Amur region (Figure 2a), the Manchu–Tungusic classification pulls it more southwards, into the area around Lake Khanka (Figure 2b).

Restricting himself to 11 Tungusic languages, notably Even, Evenki, Negidal, Oroqen, Naykhin Nanai, Oroch, Orok, Olcha, Udehe, Manchu and Xibo, Wichmann (personal communication) situates the Tungusic homeland around the location of Naykhin Nanai at latitude 49.28 and longitude 136.47. As more southern varieties such as Solon on the river Nonni in Inner Mongolia and Bikin Nanai in the southern part of the Primorye province as well as Hezhe in Heilongjiang and Jilin are lacking from this analysis, we may expect the real diversity hotspot to be more to the south than the inferred one. In sum, the diversity hotspot principle leaves room for both the Mid Amur region and the area around Lake Khanka but excludes the Baikal and the Yalu river region as plausible homelands.

The breakup of proto-Tungusic took place relatively recently. Different linguistic dating methods, such as lexicostatistics (Korovina 2011: 600 BCE), dating of ethnonym shift (Robbeets 2015: 206 BC to AD 220), measurement of mutual intelligibility (Pevnov 2012: AD 1), Bayesian phylolinguistics (Oskolskaya *et al*. (under review): AD 200), cultural reconstruction (Janhunen 2012: 500 BC to AD 500) and the automated similarity judgment program (Holman *et al*. 2011: AD 681) yield different dates, but all dates range between 600 BC and AD 700, which supports a relatively shallow time-depth for the family.

Applying cultural reconstruction to the Tungusic languages, we find that Proto-Tungusic culture can be associated with the reconstruction of PTg *sele ‘iron’, reflected in corresponding forms in the contemporary languages all sharing the meaning ‘iron’; see (1) in Supplementary Table S1. The first uncontested findings of iron in the Russian Far East go back to the Krounovska culture (600 BC to AD 200), which marks the beginning of the Early Iron Age in the region. From there, iron spread northwards, to the northern Primorye and the Priamurye during the ensuing period of the Pol'tse and Ol'ga cultures (200 BC to AD 500), but there is no evidence for iron as north as the Upper Amur region around that time. Iron reached Liaoning and southwestern Jilin no later than the third century BCE. Broadly speaking iron begins to appear early first millennium BCE in the Baikal region. Thus, the reconstruction of iron is compatible with all locations, except the Mid and Upper Amur region.

As shown in the etymologies (2)–(6) in Supplementary Table S1, we can reconstruct agricultural vocabulary to Proto-Tungusic, such as *pisi-ke ‘broomcorn millet (*Panicum miliaceum*)’, *jiya ‘foxtail millet (*Setaria italica*)’, *üse- ~ *üsi*- ‘to plant’, *üse ~ *üsi* ‘seed, seedling’, *üsin ‘field for cultivation’ and *tari- ‘to sow, plant, cultivate’. The use of these words by the speakers of Proto-Tungusic implies that they were familiar with agriculture. In addition to this familiarity with agricultural concepts, there are a number of other reasons to assume that the speakers of Proto-Tungusic were cultivating plants. First, the derivation of the Proto-Tungusic word *üse ~ *üsi* ‘seed, seedling’ as a deverbal noun from the verb *üse- ~ *üsi*- ‘to plant’ suggests that seeds were not just collected for consumption but that they were planted as part of a cultivation process.

Second, for some crop names such as ‘barley’ and ‘broomcorn millet’, we can argue that the word refers to the domesticated crop rather than to the wild variety of the plant because both crops are not native to the region and have been imported as domesticated crops. It is commonly assumed that broomcorn millet has been imported from the West Liao River region by the people who introduced the Zaisanovska culture (3200–1300 BCE) to the area around Lake Khanka (Sergusheva and Vostretsov 2009; Leipe *et al*. 2019; Li *et al*. 2020). That the origins of the agricultural vocabulary in Proto-Tungusic go back as early as the fourth millennium BC in the southern part of the Primorye is further supported by the external etymologies of some words. Since some of the terms such as *tari- ‘to sow, to plant’ have cognates in Turkic and Mongolic; these agricultural terms must have been present in proto-Tungusic since its separation from proto-Altaic, estimated at 3300 BC (Robbeets and Bouckaert 2018; Robbeets *et al*. 2020). Only in the Zaisanovska culture around Lake Khanka and in the Wangjiacun site in Dalian on the North Korean border does agriculture go back that deep in time. Thus, the presence of agricultural vocabulary excludes the Baikal region and the Mid and Upper Amur region as plausible homelands.

The rather large time difference between the separation of Proto-Tungusic from Proto-Altaic around 3300 BC and the actual dispersal of Tungusic languages after 600 BC does not imply that proto-Tungusic continued as a single language without any diversification during three millennia. On the contrary, it is safe to assume that the contemporary Tungusic languages represent but a tiny proportion of the original diversity and that in the first millennia BCE various sister languages of Proto-Tungusic were spoken in the area. The adoption of agriculture involved a previously unparallelled demographic growth in certain production areas in North East China. With its relatively mild climate, the protected region of Lake Khanka constituted such an area, close to the northern edge of the climate range for East Asian Neolithic agriculture. Demographically, this implies a large population, while linguistically, it means that speech communities which happened to be in the center of agricultural innovation underwent rapid growth and were able to absorb neighboring speech communities by way of language shift. In this way, certain Proto-Tungusic speech communities in the region became highly expansive at the expense of others. This expansion must have affected a large number of individual languages belonging to various other language families, such as the Amuric family that was reduced to only one surviving language, Nivkh, but it may also have affected now-extinct sister branches of Proto-Tungusic. From the number of surviving contemporary languages, it is clear that the Transeurasian languages in general have a relatively high extinction rate. By way of comparison, Austronesian has 1500 surviving languages, whereas hardly 50 Transeurasian languages have survived to the present. This supports the assumption that only a happy few expansive languages survived in Northeast Asia, at the expense of numerous others.

A location around Lake Khanka for the Proto-Tungusic speech community is further corroborated by contact linguistics. The term for ‘barley and similar crops’, PTg *murgi, is probably borrowed from an Old Chinese donor word, 來*mə.rˤək > *mə.rˤə, ‘a kind of wheat’ (Robbeets 2017: 28–29). The linguistic reconstruction can be correlated to the archaeological evidence for barley being first imported through Chinese contact at the time of the Krounovska culture situated in the Southern Primorye south of Lake Khanka (Sergusheva and Vostretsov 2009: 214–215; Leipe *et al*. 2019: 12).

Proceeding to the reconstruction of ecological vocabulary, we can reconstruct tree names to Proto-Tungusic, such as *inʹŋamu-kta ‘cloudberry (*Rubus chamaemorus*)’, *xiba-gda ‘ash tree (*Fráxinus mandshurica*)’, *xami-gda ‘poplar (*Populus maximowiczii*)’, *kilden ‘linden (*Tilia amurensis*)’ and *molon ‘maple (*Acer ukurunduense*)’, as shown in Supplementary Table S1 (7)–(11). Except for ‘cloudberry’, none of these species appears in the Baikal region, while maple does not appear in the Mid and Upper Amur region. Among the Proto-Tungusic tree names, the terms for ‘pine tree’ in Supplementary Table S1 (12)–(14) are particularly telling. In contrast to the wide distribution of the ‘creeping pine’, that of the ‘Korean pine (*Pínus koraiénsis*)’ is limited to the area around Lake Khanka. In addition, it is the only plant for which we have archaeobotanical evidence. The nutshells of this pine were found in pit-dwelling deposits in the Southern Primorye, from 1300 BC onwards (Sergusheva and Vostretsov 2009: 213). Therefore the reconstruction of the word for Korean pine strongly supports a homeland around Lake Khanka.

Table 1 assesses the different homeland proposals from an archaeolinguistic viewpoint. The hypothesis of Lake Khanka is best supported by the linguistic data, while the Baikal hypothesis is the least compatible. Situating the Proto-Tungusic homeland in the Lake Khanka area is further supported by the continuity of political formations in the region such as the Mohe (AD 500–1000) in the area around Lake Khanka, the Bohai state (AD 698–925) southwards from lake Khanka into present-day North Korea and the Jin Empire of the Jurchen (AD 1115–1234) to the west of Lake Khanka in the Sungari, Nonni and Middle Amur region. Since Neolithic times, the cultural edge in the Russian Far East was in the south, not in the north. Therefore, the area around Lake Khanka is the most plausible homeland for the ancestral Tungusic speech community.

**Table 1.** Evaluating the four homeland proposals for Proto-Tungusic using different archaeolinguistic techniques: 0 implausible, 1 plausible

Proposed homelands	Diversity hotspot	‘Iron’	Agricultural cognates	Loan word ‘barley’	Trees	Korean pine
1. Baikal	0	1	0	0	0	0
2. Mid and Upper Amur River	1	0	0	0	0	0
3. Lake Khanka	1	1	1	1	1	1
4. Yalu River	0	1	1	0	1	0

### Genetic results

From a paternal perspective, Tungusic speakers are associated with the Y chromosomal haplogroup C3-M217. The haplogroup C3c-M48 is prevalent in Evenki and Even, as well as in other Tungusic-speaking populations in the Amur River Basin, including Oroqen, Olcha, Negidal, Udehe and Nanai with frequencies ranging from about 20% to even 100%. The C3-M217 type also reaches high frequencies in populations surrounding the Amur River Basin, such as Nivkh (38%) and Ainu (12.5–25%) (Wei 2011). The current geographic and genetic diversity distribution pattern suggests that the dispersal of Y chromosomal haplogroup C3-M217 was most likely associated with Tungusic-related population expansion from the Amur River.

**Figure 3.**
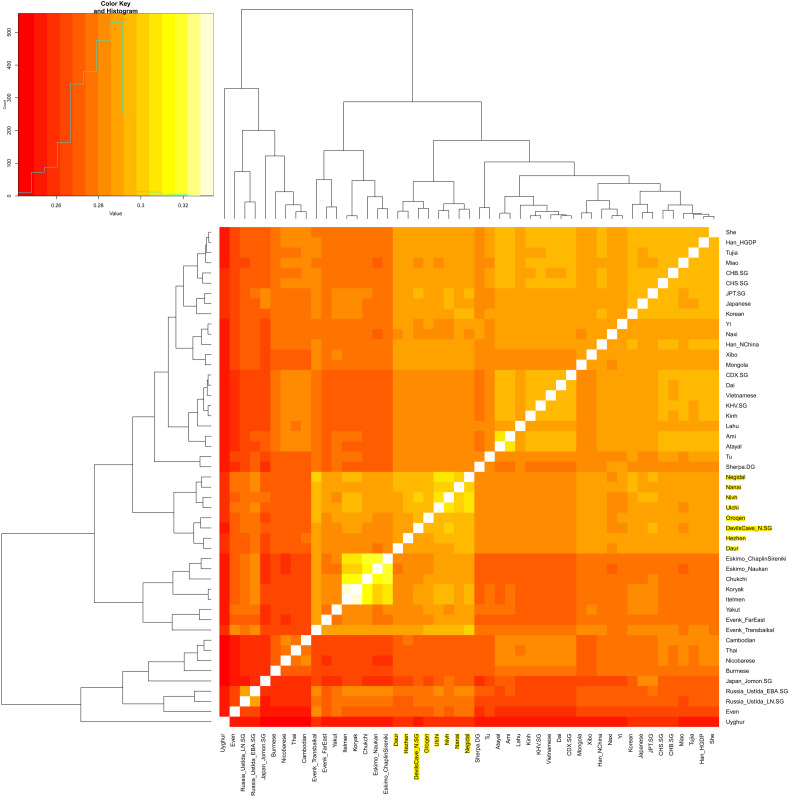
Outgroup *f*_3_-statistics of the form *f*_3_ (Mbuti; X, Y) to test the genetic drift sharing between ancient Devil's Gate and present-day East Eurasians.

From a genome-wide perspective, we reanalyzed the data of ancient samples from Devil's Gate (Chertovy Vorota) Cave dating to ~7.7 kya in the Primorye Region of the Russian Far East (Siska *et al*. 2017; de Barros Damgaard *et al*. 2018). We observe that ancient Devil's Gate samples cluster together with present-day Tungusic-speaking populations and Ulchi in the outgroup *f*_3_-statistics, as shown in Figure 3. We find a genetic substructure within Tungusic-speaking populations. The North Tungusic Evenki and Even cluster with surrounding Siberian populations other than with Amur Tungusic groups. The substructure has been caused by the fact that North Tungusic Evenki and Even have received gene flow from West Eurasian-related populations.

**Figure 4.**
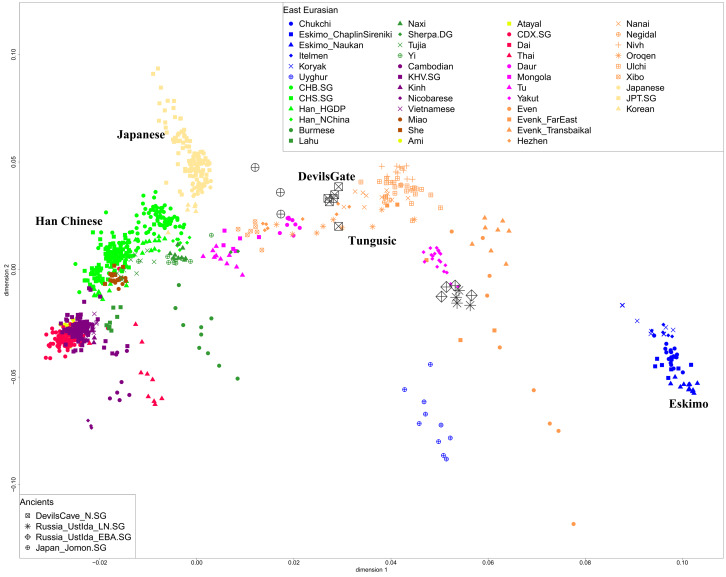
Principal component analysis projecting ancient Devil's Gate, Ustlda and Jomon samples onto the variation of present-day East Eurasians.

Our principal component analysis in Figure 4 shows that the population structure in East Asia is strongly correlated with geographical and linguistic categories. The ancient Devil's Gate genomes are shown to be genetically most similar to contemporary Tungusic populations, such as Ulchi, Negidal and Nanai at the lower Amur River basin, as well as Nivkh people on the nearby Sakhalin island, documenting a continuous presence of this type of ancestry in the Amur River Basin stretching back at least 8000 years. The principal component analysis plot in Figure 4 shows that Evenks and Evens are shifted towards West Eurasians and they have about 14–35% West Eurasian-related ancestry estimated by *qpAdm* method (Supplementary Table S2). The admixture time estimated by LD decay suggests a very recent time of around four to six generations ago (Supplementary Table S3). Late Neolithic and Early Bronze Age ancient samples from Ustlda site near the Lake Baikal region show a similar genetic profile with Even people deriving a large amount of Devil's Gate-related ancestry and also having admixture from West Eurasians.

**Figure 5.**
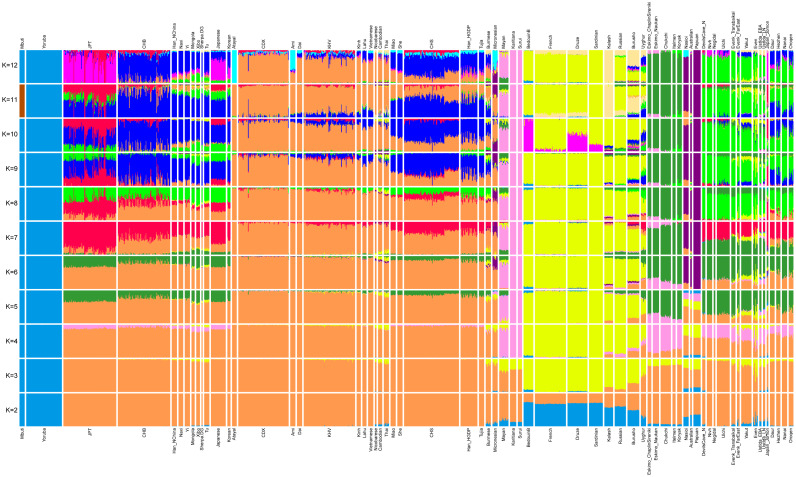
Admixture plot from *K* = 2 to *K* = 12.

The ADMIXTURE analysis in Figure 5 also confirms the above observation. We observed a large scale of shared components from ancient Devil's Gate samples to present-day Tungusic-speaking populations. We have not found evidence of any West Eurasian-related admixture in ancient Devil's Gate samples. In contrast, the Baikal Ustlda hunter-gatherers (~4700 years ago) were reported to largely have a Devil's Gate-related component, but also have West Eurasian-related ancestry as is shown in the ADMIXTURE plot.

Therefore, our genetic findings suggest that the Tungusic-related Amur ancestry distributed in a vast region from the Russian Far East to Lake Baikal. The ancient Devil's Gate population seems to be an unadmixed form of this type of ancestry, which tends to support the larger Amur River region as the ancestral homeland for present-day Tungusic-speaking populations.

## Discussion

Aligning the evidence, we find that our linguistic and genetic results converge in outlining the broader Amur River region as the ancestral homeland of the speakers of Proto-Tungusic, but our archaeolinguistic results more specifically point to the area around Lake Khanka in the southern part of the Primorye. The Amur-like genetic profile displays a deep history, going back at least 8000 years and covering an extensive region from Baikal and Liao River in the west to the Sea of Japan in the East. Our archaeolinguistic results suggest a dispersal route for agriculture and language from the West Liao River Region to the Southern Primorye around 3300 BCE. As the proposed farming/language dispersal falls within the geographical and chronological reach of the Amur-like genetic profile, we would not expect to observe traces of genetic admixture even if population movement was involved, because the admixing of two similar Amur-like genomes would not be perceivable. This explains why in the southern Primorye both Tungusic-speaking populations and non-Tungusic speaking populations such as the Nivkh are genetically similar and continuous with the ancient Devil's Gate genome in the region. Bringing the archaeolinguistic and genetic evidence together thus makes a case for imperceptible genetic admixture and indicates that the area around Lake Khanka in the southern part of the Primorye is the most plausible homeland for the ancestral speakers of Tungusic.

## Supplementary material

For supplementary material accompanying this paper visit http://dx.doi.org/10.1017/ehs.2020.8. click here to view supplementary material

## Data availibility statement

The linguistic data underlying this article are made available in the Supplementary Material (1). The genetic data are accessible through the following URL: https://reich.hms.harvard.edu/downloadable-genotypes-present-day-and-ancient-dna-data-compiled-published-papers

## References

[ref1] Alexander DH, Novembre J and Lange K (2009) Fast model-based estimation of ancestry in unrelated individuals. Genome Research 19(9), 1655–1664. 19648217 10.1101/gr.094052.109 PMC2752134

[ref2] Benzing J (1955) Die tungusischen Sprachen: Versuch einer vergleichenden Grammatik. In Abhandlungen der geistes- und sozialwissenschaftlichen Klasse 1955(11). Wiesbaden: Akademie der Wissenschaften und der Literatur in Mainz in Kommission bei Franz Steiner Verlag.

[ref2a] Chang CC, Chow CC, Tellier LC, Vattikuti S, Purcell SM and Lee JJ (2015) Second-generation PLINK: rising to the challenge of larger and richer datasets. Gigascience 4(1), 7. 25722852 10.1186/s13742-015-0047-8 PMC4342193

[ref3] Cincius VI (1949) Sravnitel'naja fonetika tunguso-man'chzhurskikh jazykov. [Comparative phonetics of the Tungus-Manchu languages]. Leningrad: Uchpedgiz.

[ref24] Damgaard PB, Marchi N, Rasmussen S, Peyrot M, Renaud G, Korneliussen T, Moreno-Mayar JV, Pedersen MW, Goldberg A, Usmanova E, Baimukhanov N, Loman V, Hedeager L, Pedersen AG, Nielsen K, Afanasiev G, Akmatov K, Aldashev A, Alpaslan A, Baimbetov G, Bazaliiskii VI, Beisenov A, Boldbaatar B, Boldgiv B, Dorzhu C, Ellingvag S, Erdenebaatar D, Dajani R, Dmitriev E, Evdokimov V, Frei KM, Gromov A, Goryachev A, Hakonarson H, Hegay T, Khachatryan Z, Khaskhanov R, Kitov E, Kolbina A, Kubatbek T, Kukushkin A, Kukushkin I, Lau N, Margaryan A, Merkyte I, Mertz IV, Mertz VK, Mijiddorj E, Moiyesev V, Mukhtarova G, Nurmukhanbetov B, Orozbekova Z, Panyushkina I, Pieta K, Smrčka V, Shevnina I, Logvin A, Sjogren KG, Štolcova T, Taravella AM, Tashbaeva K, Tkachev A, Tulegenov T, Voyakin D, Yepiskoposyan L, Undrakhbold S, Varfolomeev V, Weber A, Wilson Sayres MA, Kradin N, Allentoft ME, Orlando L, Nielsen R, Sikora M, Heyer E, Kristiansen K and Willerslev E (2018) 137 ancient human genomes from across the Eurasian steppes. Nature 557(7705), 369–374. 29743675 10.1038/s41586-018-0094-2

[ref4] Derevyanko AP (1976) Priamur'ye v drevnosti (do nachala nashej ery). [Priamur'e in antiquity. One thousand B.C.]. Novosibirsk: Nauka.

[ref5] Doerfer G (1978) Classification problems of Tungus. In M Weiers (ed.), Beiträge zur nordasiatischen Kulturgeschichte (pp. 1–26). Wiesbaden: Otto Harrassowitz.

[ref6] Georg S (2004) Unreclassifying Tungusic. In C Naeher, G Stary and M Weiers (eds), Proceedings of the First International Conference on Manchu-Tungus Studies. 2: Trends in Tungusic and Siberian Linguistics (Tunguso-Sibirica 9) (pp. 45–57). Wiesbaden: Otto Harrassowitz.

[ref7] Heggarty P (2015) Prehistory through language and archaeology. In C Bowern and B Evans (eds), The Routledge Handbook of Historical Linguistics (pp. 598–626). London: Routledge.

[ref8] Helimski E (1985) Samodiisko-tungusskie leksicheskie sviazi i ikh etno-istoricheskie implikatsii. [Samoyed-Tungusic lexical relations and their ethno-historical implications]. In EI Ubriatova (ed.), Uralo-Altaistika (pp. 206–213). Novosibirsk: Nauka.

[ref9] Holman EW, Brown CH, Wichmann S, Müller A, Velupillai V, Hammarström H, Sauppe S, Jung H, Bakker D, Brown P, Belyaev O, Urban M, Mailhammer R, List J-M and Egorov D (2011) Automated dating of the world's language families based on lexical similarity. Current Anthropology 52(6), 841–875.

[ref10] Janhunen J (1996) Manchuria: an ethnic history (Mémoires de la Société Finno-Ougrienne 222). Helsinki: Suomalais-Ugrilainen Seura.

[ref11] Janhunen J (2012) The expansion of Tungusic as an ethnic and linguistic process. In AL Malchukov and LJ Whaley (eds), Recent Advances in Tungusic Linguistics (Turcologica 89) (pp. 5–16). Wiesbaden: Otto Harrassowitz.

[ref12] Jeong C, Balanovsky O, Lukianova E, Kahbatkyzy N, Flegontov P, Zaporozhchenko V, Immel A, Wang CC, Ixan O, Khussainova E, Bekmanov B, Zaibert V, Lavryashina M, Pocheshkhova E, Yusupov Y, Agdzhoyan A, Koshel S, Bukin A, Nymadawa P, Turdikulova S, Dalimova D, Churnosov M, Skhalyakho R, Daragan D, Bogunov Y, Bogunova A, Shtrunov A, Dubova N, Zhabagin M, Yepiskoposyan L, Churakov V, Pislegin N, Damba L, Saroyants L, Dibirova K, Atramentova L, Utevska O, Idrisov E, Kamenshchikova E, Evseeva I, Metspalu M, Outram AK, Robbeets M, Djansugurova L, Balanovska E, Schiffels S, Haak W, Reich D and Krause J (2019) The genetic history of admixture across inner Eurasia. Nature Ecology and Evolution 3(6), 966–976. 31036896 10.1038/s41559-019-0878-2 PMC6542712

[ref13] Kanzawa-Kiriyama H, Kryukov K, Jinam TA, Hosomichi K, Saso A, Suwa G, Ueda S, Yoneda M, Tajima A, Shinoda KI, Inoue I, Saitou N (2016) A partial nuclear genome of the Jomons who lived 3000 years ago in Fukushima, Japan. Journal of Human Genetics 62(2), 213–221. 27581845 10.1038/jhg.2016.110 PMC5285490

[ref14] Kormušin IV (1998) Udyhejskij (udegejskij) jazyk. [The Udihe language.] Moscow: Nauka.

[ref15] Korovina E (2011) Leksika prirodnogo i kul'turnogo okruženiya v Tunguso-Man’čžurskich jazykach (v istoriko-tipologičeskom osveščenii). [Tungus-Manchu vocabulary related to natural environment and cultural activities (in historical and typological perspective).] Moscow: Russian State University of Humanities, BA dissertation.

[ref16] Lazaridis I, Patterson N, Mittnik A, Renaud G, Mallick S, Kirsanow K, Sudmant PH, Schraiber JG, Castellano S, Lipson M, Berger B, Economou C, Bollongino R, Fu Q, Bos KI, Nordenfelt S, Li H, de Filippo C, Prüfer K, Sawyer S, Posth C, Haak W, Hallgren F, Fornander E, Rohland N, Delsate D, Francken M, Guinet JM, Wahl J, Ayodo G, Babiker HA, Bailliet G, Balanovska E, Balanovsky O, Barrantes R, Bedoya G, Ben-Ami H, Bene J, Berrada F, Bravi CM, Brisighelli F, Busby GB, Cali F, Churnosov M, Cole DE, Corach D, Damba L, van Driem G, Dryomov S, Dugoujon JM, Fedorova SA, Gallego Romero I, Gubina M, Hammer M, Henn BM, Hervig T, Hodoglugil U, Jha AR, Karachanak-Yankova S, Khusainova R, Khusnutdinova E, Kittles R, Kivisild T, Klitz W, Kučinskas V, Kushniarevich A, Laredj L, Litvinov S, Loukidis T, Mahley RW, Melegh B, Metspalu E, Molina J, Mountain J, Näkkäläjärvi K, Nesheva D, Nyambo T, Osipova L, Parik J, Platonov F, Posukh O, Romano V, Rothhammer F, Rudan I, Ruizbakiev R, Sahakyan H, Sajantila A, Salas A, Starikovskaya EB, Tarekegn A, Toncheva D, Turdikulova S, Uktveryte I, Utevska O, Vasquez R, Villena M, Voevoda M, Winkler CA, Yepiskoposyan L, Zalloua P, Zemunik T, Cooper A, Capelli C, Thomas MG, Ruiz-Linares A, Tishkoff SA, Singh L, Thangaraj K, Villems R, Comas D, Sukernik R, Metspalu M, Meyer M, Eichler EE, Burger J, Slatkin M, Pääbo S, Kelso J, Reich D and Krause J (2014) Ancient human genomes suggest three ancestral populations for present-day Europeans. Nature 513(7518), 409–413. 25230663 10.1038/nature13673 PMC4170574

[ref17] Leipe C, Long T, Sergusheva EA, Wagner M and Tarasov PE (2019) Discontinuous spread of millet agriculture in eastern Asia and prehistoric population dynamics. Science Advances 5(9), eaax6225. 31579827 10.1126/sciadv.aax6225 PMC6760930

[ref18] Li T, Ning C, Zhushchikhovskaya IS, Hudson M and Robbeets M (2020) Millet agriculture dispersed from Northeast China to the Russian Far East: integrating archaeology, genetics, and linguistics. Archaeological Research in Asia 22, 100177. doi:10.1016/j.ara.2020.100177.

[ref19] Loh PR, Lipson M, Patterson N, Moorjani P, Pickrell JK, Reich D and Berger B (2013) Inferring admixture histories of human populations using linkage disequilibrium. Genetics 193(40), 1233–1254. 23410830 10.1534/genetics.112.147330 PMC3606100

[ref20] Menges KH (1968) Die tungusischen Sprachen. Tungusologie. Handbuch der Orientalistik [I. Abteilung, 5. Band: Altaistik, 3. Abschnitt] (pp. 21–25). Leiden: Brill.

[ref21] Oskolskaya S, Robbeets M and Koile E (under review) A Bayesian approach to Tungusic classification

[ref22] Patterson N, Price AL and Reich D (2006) Population structure and eigenanalysis. PLoS Genetics 2(12), e190. 17194218 10.1371/journal.pgen.0020190 PMC1713260

[ref23] Patterson N, Moorjani P, Luo Y, Mallick S, Rohland N, Zhan Y, Genschoreck T, Webster T and Reich D (2012) Ancient admixture in human history. Genetics 192(3), 1065–1093. 22960212 10.1534/genetics.112.145037 PMC3522152

[ref25] Pevnov AM (2012) The problem of localization of the Manchu-Tungusic homeland. In AL Malchukov and LJ Whaley (eds), Recent Advances in Tungusic Linguistics (Turcologica 89) (pp. 17–40). Wiesbaden: Otto Harrassowitz.

[ref26] Pictet A (1859) Les origines indo-européennes ou les aryas primitifs: essai de paléontologie linguistique (première partie, seconde partie). Paris: Joël Cherbuliez.

[ref27] Robbeets M (2015) Diachrony of verb morphology. Japanese and the Transeurasian languages (Trends in Linguistics 291). Berlin: Mouton-De Gruyter.

[ref28] Robbeets M (2017) The language of the Transeurasian farmers. In M Robbeets and A Savelyev (eds), Language Dispersal Beyond Farming (pp. 93–121). Amsterdam: Benjamins.

[ref29] Robbeets M and Bouckaert R (2018) Bayesian phylolinguistics reveals the internal structure of the Transeurasian family. Journal of Linguistic Evolution 3(2), 145–162.

[ref30] Robbeets M, Janhunen J, Savelyev A and Korovina E (2020) The homelands of the individual proto-languages: where, what and when? In M Robbeets and A Savelyev (eds), The Oxford Guide to the Transeurasian Languages (pp. 754–771). Oxford: Oxford University Press. DOI: 10.1093/oso/9780198804628.003.0044.

[ref31] Sapir E (1916) Time Perspective in Aboriginal American Culture: A Study in Method (Canada Department of Mines, Geological Survey, Memoir 90. Anthropological Series, 13). Ottawa: Government Printing Bureau.

[ref32] Sergusheva EA and Vostretsov YE (2009) The advance of agriculture in the coastal zone of East Asia. In A Fairbairn and E Weiss (eds), From Foragers to Farmers: Papers in Honour of Gordon C. Hillman (pp. 205–219). Oxford: Oxbow Books.

[ref33] Shuchardt H (1912) Sachen und Wörter. Revue Internationale d'Ethnologie et de Linguistique 7, 827–839.

[ref34] Siska V, Jones ER, Jeon S, Bhak Y, Kim HM, Cho YS, Kim H, Lee K, Veselovskaya E, Balueva T, Gallego-Llorente M, Hofreiter M, Bradley DG, Eriksson A, Pinhasi R, Bhak J and Manica A (2017) Genome-wide data from two early Neolithic East Asian individuals dating to 7700 years ago. Science Advances 3(2), e1601877. 28164156 10.1126/sciadv.1601877 PMC5287702

[ref35] Sunik OP (1959) Tunguso-man'chzhurskie jazyki. [Tungus-Manchu languages.] In Mlado-pis'mennye jazyki narodov SSSR (pp. 318–351) Moscow-Leningrad: Izdatel'stvo Akademii nauk SSSR.

[ref36] Vasilevich G (1960) K voprosu o klassifikacii tunguso-man’čžurskih jazykov. [About the classification of Tungus–Manchu languages.] Voprosy jazykoznanija, 2 [Linguistic questions, 2], (pp. 43–49). Moscow: Nauka.

[ref37] Vovin A (1993) Towards a new classification of Tungusic languages. Eurasian Studies Yearbook 65, 99–113.

[ref38] Wei LH (2011) Genetic evidences are against a common origin of the Altaic populations. Communication on Contemporary Anthropology 5, 229–236/e38.

[ref39] Whaley L and Oskolskaya S (2020) The classification of the Tungusic languages. In M Robbeets and A Savelyev (eds), The Oxford Guide to the Transeurasian Languages (pp. 82–91). Oxford: Oxford University Press. DOI: 10.1093/oso/9780198804628.003.0007.

[ref40] Wichmann S, Müller A and Velupillai V (2010) Homelands of the world's language families. A quantitative approach. Diachronica 27(2), 247–276. doi 10.1075/dia.27.2.05wic

