# Supplementary material for: The homeland of Proto-Tungusic inferred from contemporary words and ancient genomes
Source: Evol Hum Sci. 2020 Apr 22;2:e8. doi: 10.1017/ehs.2020.8 (PMC10427446; doi:10.1017/ehs.2020.8)
Supplement: Supplementary file 1 [file S2513843X20000080sup001.zip › 11_EHS_Wang&Robbeets_SI Table 2_CW.docx]

**The homeland of Proto-Tungusic**

**inferred from contemporary words and ancient genomes**

Chuan-Chao Wang & Martine Robbeets

Supplementary Table S2

**Table S3.** West Eurasian-related admixture proportions estimated using *qpAdm*. Here “p” refers to the P-value for rank=1 and “std.err” is the standard error estimated using jackknife. We used *qpAdm* to estimate West Eurasian ancestry in present-day Amur River Basin populations using Mbuti.DG, Ust_Ishim.DG, Russia_Kostenki14, Papuan.DG, Australian.DG, Onge.DG as outgroups and Han.DG and French as proxies for the source populations.

| population | p | proportion | | std.err |
| --- | --- | --- | --- | --- |
|  |  | East Asian | West Eurasian |  |
| Nivkh | 0.700 | 0.977 | 0.023 | 0.019 |
| Negidal | 0.238 | 0.970 | 0.030 | 0.022 |
| Nanai | 0.655 | 0.956 | 0.044 | 0.018 |
| Hezhen | 0.974 | 0.954 | 0.046 | 0.017 |
| Daur | 0.489 | 0.946 | 0.054 | 0.015 |
| Xibo | 0.930 | 0.929 | 0.071 | 0.016 |
| Oroqen | 0.294 | 0.928 | 0.072 | 0.017 |
| Evenk_Transbaikal | 0.528 | 0.862 | 0.138 | 0.018 |
| Evenk_FarEast | 0.865 | 0.831 | 0.169 | 0.019 |
| Even | 0.112 | 0.649 | 0.351 | 0.015 |
