# Supplementary material for: The homeland of Proto-Tungusic inferred from contemporary words and ancient genomes
Source: Evol Hum Sci. 2020 Apr 22;2:e8. doi: 10.1017/ehs.2020.8 (PMC10427446; doi:10.1017/ehs.2020.8)
Supplement: Supplementary file 1 [file S2513843X20000080sup001.zip › 10_EHS_Wang&Robbeets_SI Table 3_CW.docx]

**The homeland of Proto-Tungusic**

**inferred from contemporary words and ancient genomes**

Chuan-Chao Wang & Martine Robbeets

Supplementary Table S3

**Table S2.** Admixture time with West Eurasian-related groups estimated by ALDER.

| population | 2-ref Z-score | 2-ref decay (generations) |
| --- | --- | --- |
| Even | 5.18 | 6.83 +/- 1.32 |
| Evenk_FarEast | 5.15 | 4.35 +/- 0.84 |
| Evenk_Transbaikal | 3.67 | 5.68 +/- 1.55 |
