# Supplementary material for: The homeland of Proto-Tungusic inferred from contemporary words and ancient genomes
Source: Evol Hum Sci. 2020 Apr 22;2:e8. doi: 10.1017/ehs.2020.8 (PMC10427446; doi:10.1017/ehs.2020.8)
Supplement: Supplementary file 1 [file S2513843X20000080sup001.zip › 09_EHS_Wang&Robbeets_SI Table S1.pdf]

The homeland of Proto-Tungusic inferred from contemporary words and ancient genomes

Chuan-Chao Wang & Martine Robbeets

Supplementary Table S1

Table S1. Reconstruction of cultural and ecological vocabulary to Proto-Tungusic based on linguistic comparison of the Tungusic languages

No	Proto-Tungusic reconstruction	Attested Tungusic words
(1)	**sele* 'iron'	Evenki *sele*, Even *hel*, Negidal *sele*, Solon *sele,* Sibe *selə*, Manchu *sele*, Jurchen *sele*, Olcha *sele*, Orok *sele*, Nanai *sele*, Oroch *sele* and Udehe *sele* 'iron'
(2)	**pisi-ke* 'broomcorn millet (*Panicum miliaceum*)'	Manchu *fisihe* ~ *fisike* 'glutinous millet, broomcorn millet (Panicum miliaceum)', *fisitun* 'a ritual vessel for offering millet; bowl for grinding millet, carved out from a piece of wood' (< *fisi* + *tetun* 'utensil'); Olcha *pikse*; Nanai *pikse* 'millet'; Kur-Urmi dialect *fisxe* 'broomcorn millet (Panicum miliaceum)'; Jurchen *fise bele* 'yellow rice; coarse rice' (*bele* 'hulled rice, edible grain')
(3)	**jiya-kta* 'foxtail millet (*Setaria italica*)'	Negidal *ja:kta* 'foxtail millet (Setaria italica), small millet,; cleaned grain; oats flour'; Solon *jakta* 'porridge, food'; Sibe *je* 'foxtail millet (Setaria italica)'; Manchu *je* 'foxtail millet (Setaria Italica); grain', Jurchen *je bele* 'millet'; Nanai *jiekte* 'foxtail millet (Setaria Italica)'; Oroch *jiekte*, *jekte*, *jiekta* 'millet', Udehe *jakta* 'foxtail millet (Setaria Italica), porridge'
(4)	**üse- ~ üsi-* 'to plant'<br><br>→ **üse~üsi* 'seed, seedling'<br>→**üsi-n* 'field for cultivation'	Evenki *ihəw-* 'to grow (of people)'; Even *isu:-* ~ *esu:-* 'to sprout, come out (of plants), blossom, grow', Negidal *isew-* 'to grow, become acclimatized (about plants); mature, grow up (about people)'; Sibe *use-* 'to sow seeds', *use* 'seed, grain', *usin* 'field, farmland'; Manchu *use-* 'to plant, seed (tr.)', *use* 'seed; insect egg', *usin* 'field for cultivation', Jurchen *use* 'seedling', *usi-in* 'field', Olcha *use* 'seed', *usun* 'field, garden'; Nanai *use* 'seed', *usĩ* 'arable field for cultivation; private garden for cultivating vegetables',Orok *usi* 'field (farm), garden', *usi-* 'to cultivate, till; hunt a bear'; Oroch *usi* 'seeds, grains', *usin* 'garden for cultivation', *usin-* 'to sow, to plant in a garden'; Udehe *uhi-* 'to sow, to plant a garden', *uhi* 'garden for cultivating plants'
(5)	**tari-* 'to sow, plant, cultivate'	Evenki *tari-* ~ *tare-* ~ *tale-* 'to sow'; Solon *tari-* 'to sow seeds; to plant; to cultivate; to grow; to disseminate; to inject; to infect, to catch a disease', *tariŋko* 'injector'; Manchu *tari-* 'to cultivate, farm; to plow'; Jurchen *tali-* ~ *tari-* 'to sow, to plant, to cultivate'; Ulcha *tari-* 'to sow, to plant'; Nanai *tari-* 'to sow seeds', *tariko* 'sowing machine', *tarici-* 'to sow (seeds) regularly'; Udehe *tali-* 'to plant a garden', *tali* 'garden for cultivating plants'
(6)	**murgi* 'barley (*Hordeum vulgare*)'	Manchu *muji* 'barley (*Hordeum vulgare*)', Sibe *muji* 'barley (Hordeum vulgare)', Olcha *muji* 'oats', Nanai *muji* 'oats', Oroch *muji* 'barley', Jurchen *miryei* 'product of agriculture', Solon *mụrgil* 'spring crops, spring-sown field'
(7)	**ingamu-kta* 'cloudberry (Rubus chamaemorus)'	Evenki *ingamukta*, *inngamukta* 'cloudberry (berry)'; *ingamukta-mkurā* 'cloudberry (plant)', Even *ịnŋamtạ* 'cloudberry', Oroch *in'amụkta* 'cloudberry', Udehe (Samarga dialect) *iŋamukta* 'cloudberry', Olcha *ịn'amụqta* 'cloudberry'
(8)	**xiba-gda* 'ash tree (Fráxinus mandshurica)'	Negidal *ịwagda* 'ash tree', Manchu *ibadan* (mō) 'ash tree', Olcha *sịwaGda* 'ash tree' Nanai *sịwaGda* 'ash tree', Oroch *iwagda* 'ash tree', Udehe *joɣda* 'ash tree'
(9)	**xami-gda* 'poplar (Populus maximowiczii)'	Manchu *amida* 'poplar', Oroch *amigda* 'poplar', Udehe *amigda* 'poplar', Nanai *xạmigda:* 'poplar'

(10)	**kilden*<br>'linden (Tilia amurensis)'	Evenki *kilden* 'linden', Oroch *´kigde* 'linden', Olcha *kigde* 'linden', Nanai *kilde:* 'linden'
(11)	**molon*<br>'maple (Acer ukurunduense)'	Evenki *monon* 'maple', Negidal *monon* 'maple', Manchu *molo mo:* 'maple', Oroch *mono* 'maple', Udehe *mono* 'maple', Ulcha *mono* 'maple', Nanai *mono:* 'maple'
(12)	**koldon*<br>'Korean pine (Pínus koraiénsis)'	Evenki (Urmi dialect near Amur region) *koldon* 'Korean pine', *koldok* 'forest of Korean pines', Negidal *koldon* 'cedar; board, scaffold', Negidal *koldomo* 'made of cedar; boards', Oroch *ko:gdo(n)* 'cedar', *koŋko ~ kuŋko ~ kuaŋko ~ kogdo* 'Korean pine (pinus koraiénsis)', Olcha *kǫldō, koldo(n)* 'cedar', *koldomo* 'made of cedar', Orok *koldo mo:n'i* 'cedar', Nanai *kǫldõ* 'cedar', *kǫldo-ma* 'made of cedar', Manchu *xǫldon* 'Siberian pine', Jurchen *huô-tò mòh* 'pine, spruce', *hondo mo* 'pine tree'<br>Most dictionaries translate this word into Russian *кедр* 'cedar' which is a general term for both Siberian and Korean pine, but by default people living in the Russian Far East mean Korean pine.
(13)	**bolgi-kta*<br>'creeping pine (Pinus pumila)'	Evenki *bolgi-kta* 'dwarf pine', *bolgi-tta* 'pine seed, willow', *bolgi-g* 'a brushwood of dwarf pines', Even *bolgị-ɣ ~ bolgị-qaɣ* 'a brushwood of dwarf pines', Negidal *bolgi-kta* 'dwarf pine (Pinus pumila)', *bolgiɣ* 'a brushwood of dwarf pines', Orok. *boɣịta ~ bo:jta* 'dwarf pine', *boɣị ~ bo:ji* 'a brushwood of dwarf pines', Oroch *boggi-kta* 'dwarf pine', *boggi-nai* 'a brushwood of dwarf pines', Udehe (Samarga dialect) *bogbö* 'dwarf pine; stocked (about plants, forest)', Olcha *boljịqta* 'a brushwood, a nut tree'
(14)	**jagda*<br>'pine tree (in general)'	Evenki *jagda* 'pine', *jagda-g* 'pine forest', Solon *jagda* 'pine', Even *jaɣde* 'pine', *jaɣda-ɣ* 'pine forest', Negidal *jagda* 'pine', *jagdaɣ* 'pine forest', Olcha *jagda* 'pine', Nanai *ja:gda* 'pine', *ja:gda-ma* 'made of pine', Manchu *ǯakḓan mo:* 'pine', Jurchen *čāb-pùh* 'forest'
